# Supplementary material for: Are carbon emissions trading and green financial instruments synergistic? -Comprehensive quantitative research based on content analysis
Source: PLoS One. 2024 Mar 7;19(3):e0298601. doi: 10.1371/journal.pone.0298601 (PMC10919601; doi:10.1371/journal.pone.0298601)
Supplement: S1 Data — (ZIP) [file pone.0298601.s001.zip › Data/地级市Z_F工作报告.zip!/╔╜╢1⁄2/║╩╘≤╩╨/└┤╫╘╣1⁄2╓┌║┼í╛┬φ┐╦╩2╛▌═°í┐_2017.docx]

各位代表：

　　现在，我代表菏泽市人民政府，向大会报告工作，请予审议。请市政协各位委员、各位列席的同志提出意见。

过去五年工作回顾

　　过去的五年，是菏泽砥砺前行、在科学发展征程上不断迈出新步伐、取得新成绩、开创新局面的五年。五年来，在中共菏泽市委的坚强领导下，全市人民开拓创新，扎实苦干，圆满完成了市十八届人大一次会议确定的各项目标任务。

　　--过去的五年，我市经济加快赶超，综合实力不断迈上新台阶。地区生产总值由1557亿元增加到2560亿元，在全省前移2个位次。'四上'企业由3801家增加到6621家，在全省前移2个位次。规模以上工业主营业务收入由3467亿元增加到7786亿元，在全省前移4个位次。规模以上工业企业资产由1415.8亿元增加到3448.9亿元。地方一般公共预算收入由111.6亿元增加到185亿元。进出口总额由178亿元增加到351亿元。存款、贷款余额由1327亿元、877亿元分别增加到3025亿元、1805亿元。社会消费品零售总额由786亿元增加到1503亿元。

　　--过去的五年，我市产业结构调整优化，发展质量效益不断提升。三次产业比由14.7:54.5:30.8调整到10.9:51.3:37.8。财政收入中税收占比达到75.2%，居全省第5位。税收中的主体税种占比由40%提高到61.9%。省级以上科技创新平台由44家增加到132家，高新技术产业产值占规模以上工业的比重由27%提高到32.7%。国税收入由82.3亿元增加到153.9亿元，在全省前移3个位次。规模以上工业企业新增1142家，入库税金由71.7亿元增加到126.5亿元，能源化工、生物医药、机电设备制造、农副产品加工等主导产业规模效益显著提升。粮食总产实现'十三连增'，新增农业标准化基地180万亩，建成'一村一品'特色村镇938个，省级农业科技园区实现县区全覆盖。现代服务业占比超过传统服务业，电子商务交易额突破千亿元。蓝天白云天数持续增加，代表山东接受国家淮河流域治污核查获得第一名。

　　--过去的五年，我市基础设施建设步伐加快，城乡面貌显著变化。常住人口城镇化率由37.8%提高到47.4%。定陶撤县设区。机场、高铁、航道、高速公路等城市重大基础设施集中规划。图书馆、火车站等一大批重点工程相继竣工。改造国省道干线公路1185公里。市区建成区面积由79.1平方公里增加到143.1平方公里，常住人口由63.3万人增加到103万人，集中供热面积由320万平方米增加到880万平方米，环卫道路保洁面积由1239万平方米增加到1890万平方米。全市园林绿地面积由6951万平方米增加到1.07亿平方米。城市污水处理厂由11座增加到23座，日处理能力由30万吨提高到70万吨。农村公路通车里程由1.9万公里增加到2.2万公里，改造窄危桥472座。农村供电线路由1.1万公里增加到2.1万公里，安全饮水人口由360万人增加到760万人。建成农村新型社区372个，实现城乡环卫一体化。

　　--过去的五年，我市各项改革深入推进，发展活力不断释放。15项国家级、136项省级改革试点顺利完成。政府机构改革积极推进。实行了行政审批'两集中、两到位'，取消、承接、调整行政审批事项406项，统一规范县区行政审批事项247项，'放管服'改革成效显著。财税金融改革力度加大，现代预算制度加快建立。公共资源市场化配置、城乡医疗保险整合、养老体制并轨、县级公立医院等改革取得创新突破。'双联双创'深入实施，村居干部经济责任审计扎实推进。

　　--过去的五年，我市公共服务全面加强，民生福祉持续提升。财政用于民生的投入由164.1亿元增加到342.2亿元。累计有168.6万人实现脱贫。全市中小学在校学生人均校舍面积由5.16平方米提高到7.62平方米，补充招聘教师1.5万名。累计新增就业32万人。城乡居民人均可支配收入分别由13380元、6088元增长到22122元、10705元。企业职工基本养老金水平由每月1469元提高到每月2352元，居民基础养老金标准由每月55元提高到每月100元，城乡低保标准由每月280元、每年1800元分别提高到每月400元、每年3000元。新增三级甲等综合医院2家，每千名人口床位数由3.41张增加到4.94张，人均预期寿命由77岁提高到79岁。全民健身运动和体育事业蓬勃发展，文化惠民工程扎实推进，公共文化服务体系日益健全。社会治安、安全生产、食品药品监管等工作不断加强，平安菏泽建设深入推进。我市相继被授予国家园林城市、全国双拥模范城、全国科技进步先进市、国家级出口林产品质量安全示范市等一批荣誉称号，提高了城市美誉度，彰显了城市影响力。

　　--过去的五年，是全市上下攻坚克难、奋力拼搏的五年，也是菏泽'科学赶超、后来居上'取得重大成效的五年。五年来，全市上下保持一张蓝图绘到底的战略定力，牢牢抓住发展第一要务不动摇，把提高质量和效益摆在突出位置，牢固树立以人为本、民生优先的执政理念，始终保持勤勉敬业、担当奉献的实干作风，把全面从严治党作为事业发展的根本保证，创出了经验，巩固了成果，为今后的长远发展奠定了基础。五年来，紧紧围绕建设人民群众满意政府，不断改进工作作风，加快转变政府职能，勇于改革创新，深化政务公开，坚持依法行政，努力提高行政效能，政府自身建设全面加强。五年来，全市人民精神振奋，不甘落后，咬定发展不放松，争先恐后创一流，通过艰辛努力和超常付出，经济社会得到又好又快发展，城乡面貌发生巨大变化，人民群众得到更多实惠，各项事业取得丰硕成果，在菏泽发展的历史上留下了浓墨重彩的一页，实现了新的历史性跨越！

2016年主要任务目标完成情况

　　2016年是我们积极适应新常态、引领新发展的重要一年。一年来，全市上下紧紧围绕加快实现'科学赶超、后来居上'的奋斗目标，大力实施'四项工程'，统筹推进稳增长、促改革、调结构、惠民生、防风险等各项工作，全面完成了市十八届人民代表大会第五次会议确定的目标任务。地区生产总值可比增长8.5%，城乡居民人均可支配收入分别增长8.6%、9.2%，进出口总额增长30%，国税收入增长26.4%，金融机构存款余额增长18.6%，规模以上工业增加值增长10%，高新技术产业产值增长11.6%，以上8项主要经济指标增速均居全省第一位。地方一般公共预算收入可比增长8.8%，固定资产投资增长13.5%，社会消费品零售总额增长11.2%。各项社会事业健康发展。

　　（一）新旧动能加速转换，发展基础更加稳固。充分挖掘发展潜力，加速传统动能释放，集中培育形成新动能，进一步夯实了基础，增添了发展优势。传统动能持续发力。四大工业主导产业快速膨胀，实现主营业务收入7061.5亿元，增长9.3%，占规模以上工业的90.7%。工业企业技改投入达到379亿元，产能利用率提高4.6个百分点。招商引资和项目建设扎实推进。全年签约项目889个，计划总投资2013亿元的596个项目开工建设。12个省重点项目完成投资88.4亿元，200个市重点项目完成投资778亿元，83个项目建成投产。返乡创业蔚然成风。健全了在外人员信息库，在外出务工人员集中的城市建立了22个返乡创业服务站，召开了多层次恳谈会，推出了一系列扶持政策，推动了劳务经济向创业经济转变。全市3.5万名在外人员返乡创业，带动就业10.5万人，成为一些新兴产业发展的重要支撑力量。电子商务加快发展。淘宝村、淘宝镇分别发展到61个、6个，均占全省的1/2；网上活跃卖家达到10.8万个，居全省第4位。电商园区发展到32个，电子商务与一二三产业的快速融合，有力带动了实体经济发展。1395家规模以上工业企业'触网'，占比达到43%。跨境电商交易额2.5亿美元，增长30%。全国首个'菜鸟县域智慧物流+'项目落户我市，'全城达'同城快递业务实现市区全覆盖。电子商务交易额实现1203亿元，增长63%。我市被批准创建全国电子商务示范市。

　　（二）质量效益不断提高，转型升级步伐加快。把提质增效作为经济发展的主攻方向，努力优化经济结构，推动了转型发展。科技创新能力明显提升。山东省科学院菏泽分院获批建设。新增省级以上众创空间、科技企业孵化器、工程实验室、企业技术中心等创新平台42家。入选国家'万人计划'2人、山东省泰山产业领军人才12人。省级以上高新技术企业新增29家，达到62家。产业结构明显优化。大力推动'个转企、小升规、规改股、股上市'，新增'四上'企业650家、占全省新增总量的1/7，规模以上企业完成规范化改制503家，新增挂牌上市企业40家。步长制药在上交所成功上市，实现了我市企业在主板上市零的突破。东明石化在全省纳税百强企业中的排名由第65位上升到第34位，纳税前100名工业企业入库税金增长19.8%。大力发展商贸物流、文化旅游等现代服务业，现代服务业占服务业的比重达到50.8%，提高1.3个百分点。大力发展现代农业，新增家庭农场、农民专业合作社等新型农业经营主体6065家，标准化生产基地面积达到420万亩。大力实施品牌战略，新认定山东省著名商标15个、山东名牌11个，新注册国家地理标志商标2件，新认证'三品一标'农产品213个。外向型经济逆势上扬。加大对优势行业的定向培育，新增有进出口实绩的企业40家，出口、进口分别完成164.2亿元、186.9亿元，分别增长20.2%、39.9%。

　　（三）城镇基础设施密集实施，综合承载能力明显增强。把基础设施作为加快城市化的重要突破口和有力抓手，实施了一批重点工程。重大基础设施加快推进。抢抓政策机遇，集中力量推进了一批影响深远的重大项目。机场配套工程开工建设，鲁南高铁菏泽段基本具备开工条件，枣菏高速公路菏泽段、德上高速公路巨野至单县段全面开工，新万福河二期和郓城新河航道进展顺利，国电四期、华润电厂二期、华电定陶电力、大唐郓城电力等纳入全省能源中长期发展规划。齐鲁工业大学菏泽校区建成投用。市区基础设施加快完善。着力拉开城市框架，提高城市的通透性和微循环能力。丹阳立交桥基本建成，昆明路、人民路、丹阳路等21条道路提升改造全面完成，西安路南延、广州路北延等8项工程按计划推进。棚户区改造取得重大突破。开工棚改8.98万户，占全省的16.8%。争取棚改政策性贷款199亿元、政策性资金35.6亿元。全市房地产库存去化周期由18.1个月减少到8.8个月。城镇基础设施加快推进。坚持城乡一体发展，新建改造县乡道路246公里，10座平原水库及12座配套水厂、1800公里供水主管网建成投用。完成改厕58万户。郓城县国家级新型城镇化试点取得阶段性成果。全市'三个市民化'新增28.5万人。

　　（四）积极深化各项改革，发展活力进一步增强。牢牢抓住重点领域改革不放松，重大改革举措落地见效。供给侧结构性改革扎实推进。坚持以供给侧结构性改革为主线，着力推进'三去一降一补'。煤炭产能压缩262万吨，炼油行业淘汰落后产能350万吨。规模以上工业企业资产负债率同比下降1.1个百分点，管理费用和财务费用分别下降2.1%和6.2%。发展短板有了明显提升。行政审批制度改革持续深化。以'放管服'改革为引领，修编了市级行政审批事项目录，取消、承接、调整行政审批事项19项，激发了大众创新创业活力。全市新登记各类市场主体18万户、增长21.8%，其中私营企业9.4万户、增长287.5%。财税金融体制改革成效凸显。'营改增'全面实施，预算管理制度进一步规范。市级新设立4只发展投资基金，总规模达26亿元。加大项目策划、储备、申报、推介力度，大力推动政府和社会资本合作。纳入国家发改委项目库的项目318个、总投资3173亿元，分别占全省的20.9%、28.5%。纳入财政部项目库的项目321个、总投资2389.8亿元，分别占全省的30.2%、24%。94个合作项目开工建设。各项改革统筹推进。中小学校长职级制和去行政化改革扎实推进。不动产统一登记制度基本建立。市区城市管理体制改革成果进一步巩固。农村土地承包经营权确权登记颁证工作通过省级验收。市县级公立医院综合改革全面启动。全市党政机关公车改革顺利完成，取消公务用车2642辆。

　　（五）着力补齐民生短板，群众满意度不断提升。坚持共享发展理念，尽心竭力改善民生，财政用于社会公共服务的投入占财政支出的比重达到79.6%，提高1.9个百分点。脱贫攻坚成效显著。积极实施'一户一案、一村一品、一人一岗'精准扶贫模式，建设扶贫车间、双向承诺机制等扶贫做法，在全国创出了经验。引导金融机构发放扶贫贷款44.8亿元，统筹整合各类涉农财政资金17.1亿元，全部用于脱贫攻坚。44.7万人实现脱贫。民生实事扎实推进。10个方面的29项民生实事，农村幸福院、早夜市、新能源公交车、城乡电商服务网络建设、集中供热和环卫保洁等13项超额完成，市民文化中心、人行天桥、提高医保最高支付限额、淘汰取缔燃煤锅炉等15项全面完成，全民健身中心部分完成。加大了义务教育投入。投入40.9亿元用于'全面改薄'，新建校舍233.1万平方米；投入42.7亿元用于化解城镇中小学'大班额'，竣工学校85所，新增教学班2662个、学位12.1万个；招录教师4481名，开工建设乡村教师周转宿舍项目118个；义务教育均衡发展实现突破，巨野县、单县通过国家验收。生态环境进一步改善。淘汰落后产能设备850台套，实现节能15万吨标煤，全面完成省下达的节能减排任务。三条主要出境河流断面水质稳定达到三类标准。全年蓝天白云天数224天，同比增加38天。

　　（六）切实加强政府自身建设，履职尽责能力进一步提高。建立学习培训制度，引领新常态发展的能力和水平不断增强。不断完善重点经济工作考核办法，导向和压力作用进一步发挥。把人代会确定的发展目标细化为243项重点工作任务，全部落实到市政府领导班子成员、责任部门和责任人，完成情况定期通过媒体向社会公开。严格按照法定权限和程序行使权力、履行职责，自觉接受市人大常委会的法律监督和工作监督，主动接受市政协的民主监督，广泛听取各民主党派、工商联和社会各界的意见，43件人大代表建议、276件政协提案全部办理。

　　妇女儿童、关心下一代、残疾人、老龄、慈善等事业加快发展，民族宗教、外事侨务、人民武装、防震减灾、邮政通讯、统计、物价、气象、人防、对台、史志、档案等工作都取得新成绩。

　　各位代表！过去的一年，面对各种困难和挑战，面对各种风险和考验，我们创新思路谋发展，心无旁骛抓落实，经济社会发展取得丰硕成果，成绩来之不易。这是省委、省政府亲切关怀、大力支持的结果，是中共菏泽市委坚强领导的结果，是市人大、市政协和社会各界有效监督、鼎力支持的结果，是全市人民辛勤付出、奋力拼搏的结果。在此，我谨代表市人民政府，向全市人民，向各级人大代表、政协委员，向各民主党派、工商联、人民团体和社会各界人士，向离退休老领导、老同志，向驻菏部队、武警官兵、公安干警，向所有关心支持菏泽建设和发展的同志们、朋友们，表示衷心的感谢并致以崇高的敬意!

　　在看到成绩的同时，我们也清醒地认识到，全市经济社会发展中还存在不少困难和问题。经济发展不充分、总量偏小仍是主要矛盾。产业层次低，创新能力不足，实体经济生产经营遇到新的困难，转型升级发展任重道远。投资后劲不足，带动力强、竞争力强的大项目少，招商引资力度亟需加大。城镇化水平低，中心城区承载带动能力弱，城镇基础设施配套不完善，城乡一体化水平不高。基本公共服务供给仍然不足，环境、交通、教育、医疗等民生工作短板明显。基层社会治理有不少差距。政府职能转型不快，新常态下组织推动经济社会发展的能力还需要进一步提高。党风廉政建设还有一些薄弱环节。对存在的这些困难和问题，我们一定高度重视，采取更加有力的措施认真加以解决。

未来五年发展展望

　　市第十三次党代会为我们描绘了未来五年的宏伟发展蓝图。今后五年，我国将全面建成小康社会。这个历史阶段，是我市抢抓机遇、乘势而上、奋发有为、聚力发展的重大战略机遇期，对我市发展具有重大的战略意义。内生动力愈发强劲。习近平总书记亲临菏泽视察，对我们寄予了'后来居上'的殷切期望，极大地振奋了全市人民的精神，激发了全市干部群众干事创业的热情，坚定了加快发展的信心和决心，全市上下人心思上、人心思干、人心思进的氛围日益浓厚。外部支持政策叠加发力。中央和省推出了一系列支持区域协调发展的政策举措。国家实施中部突破战略，与中原经济区、中原城市群、西部经济隆起带等政策相互叠加，我市在财税、金融、投资、人才等方面将得到更有力的支持。重大基础设施加速完善。机场、高铁、铁路、高速公路、航道等一大批重大基础设施工程集中开工建设，我市综合承载能力将显著提升，承东启西、引南连北的区位优势将进一步显现出来。城市带动能力持续增强。我市作为全省最年轻的地级市，基础设施配套、公共服务设施建设、棚户区改造规模空前，发展潜力巨大，将有力带动经济发展。主导产业逐步壮大。五大主导产业规模不断膨胀、结构持续优化，企业平均注册成立时间短，设备新、产品新、工艺先进，企业的综合竞争实力较强，产业支撑更加有力。新生动能加速形成。'城归'使新人口红利在市内形成，互联网经济引领带动能力逐步放大，特色小镇加快建设，对外贸易快速增长，新产业、新业态、新模式不断涌现，动力引擎更加强劲，推动形成后发优势。脱贫攻坚全面完成。期间将汇集大量资金、人才、项目等发展要素，加快补齐短板，有力推动协调发展。

　　今后五年里，我市将跨入更好更快发展时期。地区生产总值、地方一般公共预算收入、规模以上工业增加值年均分别增长8.5%、9%和9.5%以上，固定资产投资、规模以上工业主营业务收入、社会消费品零售总额、进出口总额等指标增速持续保持全省前列，经济总量大幅增加，综合实力在全省位次实现攀升。工业主导地位更加突出，高新技术产业产值占规模以上工业的比重、服务业增加值占三次产业的比重等指标达到或接近全省平均水平，一二三产业融合发展，经济结构明显优化，发展质量和效益显著提升。

　　今后五年里，我市将全面实现小康。脱贫攻坚夺取全面胜利，现有贫困群众全部脱贫，稳定实现不愁吃、不愁穿，义务教育、基本医疗和住房安全有保障。城乡居民人均可支配收入增幅分别保持在8.5%和9%以上，收入水平不断提升，生活质量持续提高。返乡创业热情充分迸发，就业、医疗、养老保障体系更加健全，义务教育实现均衡发展，城乡文体设施日益完善，社会化集中供水实现城乡全覆盖，农村硬化道路实现'户户通'，互联网服务全面融入群众生活，人民群众的获得感、幸福感大幅提升。

　　今后五年里，我市将迎来撤地设市20周年。以此为契机，加快城市化步伐，提升城市化水平，加快打造富有吸引力和影响力的区域中心城市。定陶区与主城区实现有机融合，发展框架进一步拉开，市区人口达到130万人以上。城市公共设施项目加快实施，城市功能更加完善。城市管理体制机制日益成熟，有效提高宜居宜业宜商宜游水平。市区、县城、小城镇、农村新型社区'四点对接'的城镇体系基本形成。常住人口、户籍人口城镇化率分别提高到54%、45%以上。

　　今后五年里，我市将跨入立体化交通时代。全市将建成1个机场、2条高铁、3条铁路、3条航道、5条高速、17条国省道，实现县县通高速公路，实现高铁在我市东西南北交汇。菏泽将由一个交通不便的四省交界城市，嬗变为以高铁引领、水陆空并行的立体式交通枢纽城市。现代化交通将助推我市成为承接产业转移的桥头堡、生产要素的集聚地，城市发展的空间将更加广阔。

　　今后五年里，我市社会发展将更加和谐稳定。生态建设扎实推进，环境保护持续强化，森林覆盖率不断提高，大气、水、土壤污染得到有效治理，空气质量良好率达到60%以上，呈现出更多的碧水蓝天。城乡环境明显改善，70%以上的乡村建成美丽乡村。社会治理更加规范，社会矛盾有序化解。'一都四乡'文化名片更加靓丽，文明创建深入推进，人民群众安居乐业。

　　各位代表，蓝图已经绘就，奋进正当其时。只要全市人民心往一处想、劲往一处使，凝心聚力，扎实苦干，勇于创新，开拓进取，就一定能够铸造菏泽发展的新辉煌，全市人民在追求幸福美好生活的征程上就一定会阔步前进！

2017年的目标任务和工作重点

　　2017年是新一届政府的开局之年，是实施'十三五'规划承前启后的重要一年，是落实市第十三次党代会精神的关键一年，做好今年的工作意义十分重大。

　　今年全市经济社会发展的主要预期目标是：地区生产总值增长8.5%左右，地方一般公共预算收入增长8.5%左右，进出口总额增长7%，城镇登记失业率控制在4%以内，城乡居民人均可支配收入分别增长9%和9.5%左右；全面完成省下达的节能减排、安全生产等约束性指标。

　　（一）突出加快发展，全力扩大优质增量，厚植发展优势。发挥有效投资的战略支撑作用，着力优化投资结构，提高投资总量，增强可持续发展后劲。一是全力抓好精准招商。毫不动摇地把招商引资作为经济工作的生命线，围绕延伸主导产业链条，高水平策划一批市场前景好、科技含量高、带动能力强的项目，争取更多纳入国家、省规划计划。瞄准产业转移的重点区域，盯紧国际国内500强和行业领军企业，灵活运用政策招商、园区招商、产业招商、以商招商等方式，不断提高招商引资的精准性和实效性，力争新引进落地过亿元项目400个以上。二是切实抓好重点工程项目建设。加快推进省市重点项目建设，确保竣工投产80个以上。加快推进重大交通项目建设。菏泽机场、枣菏高速公路菏泽段、德上高速公路巨野至单县段进度加快，新万福河航道复航工程二期开工建设，鲁南高铁菏泽段全面启动，京九铁路客运专线、菏徐铁路、濮阳至菏泽高速公路等前期工作取得新进展，东新高速东明黄河公路大桥实现贯通。加快推进公共设施项目建设。启动建设菏泽国际会展中心、电子商务综合服务中心、牡丹健康城、市博物馆新馆、市档案馆新馆、武警支队三位一体等项目。加快建设山东省科学院菏泽分院、全民健身中心、老干部活动中心、老年大学、市民文化中心、残疾人康复中心。三是强化民间投资的支撑作用。精心抓好政府和社会资本合作项目的策划、论证和筛选，争取更多项目进入国家发改委和财政部综合信息平台、纳入国家示范项目库。以政府和社会资本合作项目为重点，放开投资领域，降低准入门槛，引导更多民间资本公平进入，提高项目规范化运作的能力和落地水平，确保实际利用民间资本1200亿元以上。四是放大政府投资引导效应。坚持市场化运作，加快投融资体系建设。市级切实建设运营好菏泽牡丹机场投资公司、高铁投资公司等6家市管投融资公司，支持各县区加快建设各具特色的融资平台。积极争取国家专项建设基金项目。

　　（二）突出转型升级，提高发展的质量和效益，积极构建高水平产业体系。坚持从供给侧和需求侧两端发力，协同推进，跨越发展。一是深入实施工业强市战略。把工业主导产业作为转型发展的重中之重，紧密对接'中国制造2025'山东行动纲要，全面实施工业强市各项政策措施，落实产业推进计划，加强企业分类指导，全力培育骨干企业，切实做大做强做优工业经济。推进产业结构优化升级。实施新一轮技术改造，促进四大工业主导产业向创新链、产业链、价值链和财税链'四链'融合转变，结构层次由中低端向中高端迈进。积极培育服务型制造，推广总集成总承包、个性化定制、装配式建筑等新业态新模式。推进产品结构优化升级。鼓励企业瞄准市场需求，提升产品质量，打造优势产品。加大商标注册和品牌创建力度，力争申报国家驰名商标2件、省著名商标15件，创建省名牌产品5个，注册马德里商标40件以上。推进组织结构优化升级。深入实施企业梯次培植计划，加快'个转企、小升规、规改股、股上市'步伐，力争新增'四上'企业350家，完成改制400家、挂牌上市10家。推进经营者结构优化升级。实施企业家培育工程。倡树企业家精神，营造尊重、关心、保护企业家的良好氛围。二是加快发展现代农业。全面落实中央一号文件精神，深入推进农业供给侧结构性改革，加快培育农业农村发展新动能，不断提高农民收入。积极发展园区农业。重点抓好国家牡丹农业科技园区、8个省级农业科技园区和省级农业高新技术开发区建设，支持各县区规划建设现代农业综合园区，鼓励有条件的乡镇打造特色农业园区，支持有条件的乡村建设田园综合体。积极发展标准农业。健全农产品质量安全全过程监管体系，开展农产品质量安全追溯试点，新增农产品质量安全县区4个。新增省级标准化生产基地15个、面积5万亩。积极发展品牌农业。新增'三品一标'产品100个、省级知名品牌企业5家。积极发展效益农业。稳妥建设'万元田'。缩减低效作物种植面积，发展设施农业和牡丹、芦笋、山药、黄桃、大蒜、木瓜、罗汉参等特色作物，新增市级以上'一村一品'示范村镇30个。积极发展加工农业。突出农产品加工业园区建设，着力引进建设一批农产品加工项目，切实提高农产品加工转化率。积极发展循环农业。推进'粮改饲'和农作物秸秆'过腹还田'，提高粮食就地转化率，新增省级以上畜禽标准化示范养殖场30家。鼓励适度规模经营，新增市级以上示范家庭农场10家，农民合作示范社30个。三是推进现代服务业集聚发展。突出抓好100个服务业重点项目、100家服务业重点企业和30家重点服务业集聚区。大力发展物流业。规划建设物联网产业园。大力发展冷链物流、仓储物流、智能物流、第三方物流等现代物流业。加快济铁物流园、传化物流园等重点项目建设，引导银田农贸、喜地冷链物流园等重点企业发展智慧仓储物流和标准化试点，加快打造区域性物流中心城市。大力发展金融业。新引进银行、保险等金融机构4家以上，积极稳妥发展互联网金融、绿色金融、金融租赁等新兴业态，引导金融机构加大对实体经济的投入。大力发展现代旅游业。支持郓城县、单县发展全域旅游，加快培育旅游新业态。集中力量打造龙头旅游项目，创建旅游品牌，实现旅游消费总额150亿元以上。大力发展新兴服务业。在提升改造批发零售、住宿餐饮等传统服务业的基础上，推动建安、会展、健康、养老、家政、文化、体育等产业繁荣发展。争创国家级广告产业园区。力争服务业增加值占三次产业的比重、现代服务业占服务业的比重均提高1个百分点以上。四是提高绿色发展水平。认真落实节能评估审查制度和煤炭减量替代工作方案，大力推进'工业绿动力'计划和'百项节能重点工程'，新建建筑一律执行绿色标准，实现节能量20万吨标煤以上。强化大气污染防治，健全联防联控机制，严格执行大气污染第三阶段排放标准限值，实施民用散煤综合治理，加强扬尘治理，加快城区裸露土地绿化，持续开展加油站清理整顿和油品质量专项检查，强化高排放车辆监管，完善重污染天气应急预案，推动空气质量持续改善。狠抓水环境治理，完善落实'河长制'，搞好市县城区污水管网铺设和城市污水处理厂扩容运行，强化城市黑臭水体整治，不断改善提升水质。推进农业面源污染治理，探索推行垃圾分类制度，倡导绿色消费模式和生活方式。加大环境保护执法力度，对环境违法行为实行联合惩治、严厉惩治。

　　（三）突出创新驱动，加快改革进程，充分释放发展活力。坚持改革创新，着力破除体制机制障碍，为发展增添新动力。一是深化供给侧结构性改革。全面落实'三去一降一补'五大任务，振兴实体经济，扩大有效供给。加快推动产能过剩企业主动退出、优化整合。有效处置'僵尸企业'，实现有效激活或市场出清。加大不合理涉企收费清理力度，落实小微企业和高新技术企业税收优惠政策，扩大直供电范围。鼓励企业开展管理对标活动，推进管理创新、商业模式创新，提升精细化管理水平，实现降本增效。二是深化重点领域改革。深化行政管理改革。加快行政审批、商事制度改革，积极推行'多证合一、一照一码'。深化财税、投融资体制改革。推进政府和社会资本合作。健全完善全口径政府预算体系。深化教育、医疗卫生、社保、文化、价格机制、收入分配、住房制度、编制人事、事业单位等领域改革，进一步推进基本公共服务均等化。深化农村产权制度改革，推动'三权分置'，加快土地流转平台建设，促进土地经营权依法自愿有偿流转，逐步增加农民的财产性收入。三是深化科技创新驱动。落实支持科技创新的政策措施，引导全社会加大科技投入，确保全社会研发投入占地区生产总值的比重达到1.44%，力争有效发明专利拥有量突破千件。加强知识产权保护，严厉打击侵权假冒行为。强化企业技术创新主体地位，支持企业建设创新平台，新增市级以上技术中心15家，规模以上工业企业设立研发机构的比重达到8%。加快与清华大学、中科院、山东大学的产学研合作，落实重大合作项目10个以上。全面落实人才政策。力争新增省级以上高新技术企业25家，高新技术产业产值实现2750亿元、占规模以上工业的比重达到33.7%。

　　（四）突出优势发挥，激发内生动力，培育壮大经济增长新动能。在提升优化传统动能的基础上，大力培育新动能。一是推进新人口红利加快释放。充分发挥150多万在外务工人员这一潜在资源优势，认真落实支持返乡创业政策，完善支撑体系，降低创业门槛，提供创业便利，形成推动返乡创业的合力，加快人力回归、资本回流，力争5万名以上外出人员变为'城归'，带动就业15万人以上。二是加快发展互联网经济。把'互联网+'贯穿到产业发展全领域，促进线上线下融合发展。以创建全国电子商务示范市为抓手，大力推进电商提质增量，完善配套产业，带动新实体经济蓬勃发展，打造鲁苏豫皖交界地区具有区域竞争力、在全国具有影响力的电子商务中心城市。继续加强与阿里巴巴、京东等电商企业合作，拓宽合作领域，取得更大成效。抓好电商人才引进培养、快递物流体系建设和融资渠道拓展。全力办好第五届中国淘宝村高峰论坛，推动我市形成电商高地。加快电子商务品牌塑造，力争全市淘宝村发展到100个。积极开展'千企触网'行动，力争50%以上的规模以上企业具有网络购销实绩。引导企业推动产品上行，力争电子商务交易额达到1600亿元。三是加快发展开放型经济。加快青岛保税港区菏泽功能区等开放型功能区建设。抓好出口企业的引进培育，加强出口品牌打造。发挥户外家具、木草柳编、化工、干鲜果蔬等产品的出口优势，增强对进出口的拉动能力。深度挖掘潜力，大力发展服务贸易，切实提高服务贸易在外贸中的占比。力争实现进出口总额376亿元，服务贸易进出口17亿元。四是提升园区经济发展水平。制定出台创新市开发区和菏泽高新区行政管理体制的意见，积极争创国家级园区。支持各县区加快省级经济开发区建设。规范提升拟保留化工园区。精心抓好现代农业综合园区、电商产业园、物联网产业园、返乡创业园、商贸物流园等产业园，促进园区经济占比不断提高。加快3个省级、13个市级特色小镇的规划建设，推动形成镇域经济新的增长点。

　　（五）突出功能完善，狠抓基础设施建设，加速推进新型城镇化进程。以建设富有吸引力和影响力的区域中心城市为目标，以纳入中原城市群发展规划为契机，以迎接撤地设市20周年和实施大城市试点为动力，加快城市基础设施建设，提升新型城镇化的质量和水平。一是着力提高中心城区首位度。加强城市规划编制。紧密结合大城市试点要求，编制大城市发展规划。完成城市总体规划报审，启动综合交通、地下空间利用等专项规划和高铁片区等控制性规划。严格规划实施，提高城市规划的实施符合率。加快城市框架体系构建。围绕拓展城市发展空间，完成广州路北延跨赵王河连接鄄城县工程，广州路南延跨万福河连接定陶区工程，北外环路东延跨赵王河、穿京九铁路连接上海路工程，西安路南延穿新石铁路、跨万福河连接定陶区工程，长江路东延至机场等6条骨干道路。开工建设太原路北延从大学路至国花大道段、解放街北延从黄河路至北外环路段等6条重点道路。切实提升市区微循环能力。打通大学路从太原路至桂陵路段、泰东路从花园路至人民路段等28条道路。开工建设北外环路东延、漓江路、万泉路西延等3座穿京九铁路桥。建成康庄路、天香路、广州路北延、北外环路东延等5座跨赵王河桥，广州路、西安路南延等2座跨万福河桥工程。完成人民路与八一路、中华路等13个重要路口的渠化改造工程。进一步完善城市配套功能。继续推进便民市场、健身广场等便民设施建设，抓好供电、供热、供气、供排水等市政设施建设。扎实抓好主街道形象提升，改造提升中华路从昆明路至西三路段、广州路从开发区界至长城路段等21条道路。开工建设魏楼水库。加快推进菏泽智能电网建设。推进生态城市建设。启动菏曹运河、牡丹生态广场等17项综合治理工程，完成七里河河道一期、万福河拓宽等14项重点工程，推进赵王河上游生态公园、赵王河下游片区综合开发工程。二是大力推进棚户区改造。抢抓棚改政策最后窗口期，加大推进实施力度，确保全市133个片区、18.2万户棚改任务顺利完成。抓好棚改资金落实，提高资金使用效率。坚持惠民利民导向，切实做到依法征收、和谐征收。抓好拆迁土地推介招商，推进成方连片综合开发，不断提升城市品位。及时开工回迁安置工程，有效降低安置成本，确保回迁安置户早日住上新房。三是提升城市综合治理水平。深化市区城市管理体制改革成果，推进城市属地化、网格化、标准化管理。实施城市精细化治理工程，精雕细琢街景容貌，持续开展背街小巷、卫生死角、护栏破损、道路坑洼、店外店、占道停车、占道经营等集中整治，着力解决环境卫生、交通秩序、小区物业、户外广告、渣土清运、噪声扰民等突出问题。集中打好治理违法建设攻坚战。启动建设历史文化名人雕塑工程。四是加快城乡一体化发展。进一步提升城乡公用设施服务水平，重点建设城市'十个系统'和农村'七改'工程。积极推进智慧城市、海绵城市建设。鼓励各县抓好县城建设，增强服务功能和集聚带动能力。抓好郓城县中等城市、东明县Ⅰ型小城市试点，加快巨野县龙堌镇等省级示范镇建设。深入实施美丽乡村建设，推进基础设施和公共服务设施向农村延伸，新建市级以上示范村100个。五是扎实推进'三个市民化'。力争实现'三个市民化'18.3万人，常住人口、户籍人口城镇化率分别提高到49%、40%以上。

　　（六）突出精准施策，科学持续发力，坚决打赢脱贫攻坚战。把脱贫攻坚作为第一民生工程，大力推进精准扶贫、精准脱贫。一是实施'三个一'脱贫攻坚工程。推进'一户一案' 精准扶贫，完善双向承诺机制，逐村逐户逐人落实帮扶方案。抓好'一村一品'产业扶贫，大力发展特色种养业、传统加工业，建立特色产业扶贫示范基地，加大乡村旅游扶贫、电商扶贫、光伏扶贫力度，实现贫困村特色产业全覆盖。深化'一人一岗'就业扶贫，加大扶贫车间建设力度，确保有条件的村实现扶贫车间全覆盖。二是强化脱贫政策保障。落实政策措施，健全投入机制，对扶贫资源精准化配置，加大财政扶贫专项资金投入，强化涉农资金整合，壮大特色产业发展、小额贷款扶贫担保、公益事业发展等扶贫基金规模，放大金融扶贫政策效应。三是突出脱贫攻坚重点。聚焦重点群体，完善社保兜底政策，着力解决因病致贫返贫等难点问题。如期完成黄河滩区脱贫迁建任务。四是健全脱贫攻坚长效机制。创新责任人帮扶贫困户方式，强化部门单位扶贫责任，深入开展与青岛市的扶贫协作。基本实现农村贫困人口'两不愁、三保障'。全面落实社会保障措施，统筹解决好城市贫困等问题。加大督查考核问责力度，促进脱贫措施有效落实。

　　（七）突出以民为本，推动共享发展，让发展成果更多更好惠及人民群众。积极回应社会关切，持续加大民生投入，着力增加公共服务有效供给，让人民群众生活逐步改善和提高，获得感、幸福感和满意度不断提升。一是提高公共服务水平。提升教育发展水平。进一步加大投入，实现各县区全部通过义务教育均衡发展验收，基本解决城镇普通中小学'大班额'问题，按计划完成农村义务教育薄弱学校改造任务。稳定农村中小学教师队伍，加强幼儿园和幼儿教师队伍建设。支持驻菏高校加快发展，推进产教融合、校企合作。加强'双师型'队伍建设，提升职业教育水平。积极发展大健康产业。基本建成分级诊疗服务体系，完善家庭医生签约服务机制。健全公立医院法人治理结构，加强卫生机构标准化建设。组织好省里免费为乡镇卫生院培养全科医生的工作。完善疾病预防控制体系。继续开展出生人口性别比综合治理，强化出生缺陷综合防治。推动公办养老机构改革，发展医养融合型养老机构，新增养老床位3500张。建立居家养老服务体系，提升居家养老水平。认真落实全民健身国家战略，建设便民惠民体育公共服务体系，启动建设市体校新校区二期工程。开展残疾人精准康复服务行动。繁荣发展文化事业。大力发展文化产业，建设好文化产业示范园区和基地。加快县级国家综合档案馆和档案信息化建设，抓好第二轮修志。完善现代公共文化服务体系，城乡社区基层综合性文化服务中心达标率提高到80%。深入挖掘优秀传统文化，做好非物质文化遗产传承保护。二是提高社会保障水平。认真落实促进就业的政策措施，广开就业渠道。推进全民参保，基本实现法定人员全覆盖。全面实施机关事业单位养老保险制度。扩大合规医疗费用范围，实现居民医疗保险市级统筹。三是提高社会治理水平。深化平安菏泽建设，完善立体化、信息化社会治安防控体系，健全社会矛盾排查预警和调处化解综合机制，加强警民联防联治。加快公共法律服务中心建设，提高公共法律服务保障。加大对民间非法集资整治力度。狠抓安全生产责任体系落实，完善风险分级管控和隐患排查治理双重预防体系。加强食品药品安全监管，启动省级食品安全城市创建。继续实施农村公路安全生命防护工程，加强农村道路管护。巩固双拥共建成果，创建省级文明城市。四是扎实办好10个方面的民生实事。（1）搞好青年湖公园改造提升，规划建设环堤公园、体育公园。（2）在市区公共区域实施无线网络免费开放，把100个村建成'为村'。（3）建设菏泽市青少年综合社会实践活动基地。（4）在市区打造10公里城市步行绿道。（5）在市区新增加新能源公交车100辆、公交线路5条，新增公共自行车1000辆、站点50座，新建公厕30个、垃圾中转站10个、停车场20个。（6）新建农村校舍100万平方米以上，新建、改扩建城区中小学70所以上。（7）为全市65岁及以上老年人建立健康档案，免费开展一次健康检查。（8）在全市完成'明厨亮灶'1000家，建成食品检测平台150个。（9）新建文化广场1000处，免费送戏下乡3000场。（10）完成811个小城镇、中心村、省定贫困村电网改造，实现7.2万眼机井通电。新建改建农村公路4500公里以上。

全面加强政府自身建设

　　新目标、新任务、新征程对政府自身建设提出了新要求。我们将牢记使命，坚守责任，以改革创新精神积极应对新挑战，以科学务实作风不断攻克新难关，努力开创政府各项工作新局面。

　　（一）坚持以坚定的信念忠诚担当。始终树牢政治意识、大局意识、核心意识、看齐意识，把对党忠诚、对国家忠诚、对人民忠诚、对事业忠诚体现到实际工作中。增强直面问题的责任担当，切实担负起推进改革发展的重任。努力以更严的要求、更高的标准推动工作，在抓落实、促发展上争一流、见实效。牢固树立服务理念，以服务推动发展、以服务促进建设。牢记政府是人民的政府，把群众观点融于血脉，把群众的评价作为衡量工作的标尺，真心实意、倾情倾力为群众办实事、做好事、解难事，让政府工作更加贴近群众，更加顺民意得民心。

　　（二）坚持以更强的本领提高效能。把学习作为一种时代责任、一种精神追求、一种工作状态，适应互联网时代和经济发展新常态，加快知识更新，优化知识结构，注重思考研究，加强实践锻炼，努力更加具备专业思维、专业素养、专业方法。强化对干部的精准培训，继续组织各级政府工作人员进行专题学习、研讨，拓展工作思路，推动工作创新，努力提高政府工作人员抓工作落实的能力，不断提高政府工作的科学性、针对性和实效性。

　　（三）坚持以严格的规范依法行政。坚决服从市委领导，自觉接受市人大法律监督、工作监督和市政协民主监督，虚心接受各民主党派监督，提高人大代表议案、建议和政协委员提案办理质量。认真落实重大行政决策法定程序，坚持公众参与、专家论证、风险评估、合法性审查、法律顾问和集体讨论决定等行政决策制度，坚持把法治精神、法治思维、法治方式融入政府工作全过程，做到授予有据、行使有规、监督有效。

　　（四）坚持以勤勉的精神踏实苦干。牢记责任使命，坚持科学谋事、勤勉干事、努力成事。对确定的重点工作和重大事项以'钉钉子'精神抓好落实。践行一线工作法，把工作的重心下沉到基层，做到情况在一线掌握、问题在一线解决、工作在一线推动。强化行政问效，建立完善全方位的责任体系和监督考核机制，严肃查处不作为、慢作为、乱作为等行为，杜绝庸懒散现象，以各级政府工作人员的辛苦指数努力换取人民群众的幸福指数。

　　（五）坚持以刚性的约束廉洁从政。始终把纪律挺在前面，把规矩记在心上，把全部心思用在干事创业上。严格遵守中央'八项规定'精神和廉洁自律准则，驰而不息纠正'四风'。严格落实全面从严治党主体责任，切实加强政府系统党风廉政建设，巩固深化作风建设成果。加大对项目建设、土地管理、公共资源交易等重点领域的监管力度，严厉查处发生在基层群众身边的腐败现象和行为。强化行政监察和审计监督，政府所有工作人员都要自觉牢固树立并形成在'玻璃房'里工作的思维方式和行为方式，做到全方位透明、全方位公开、全方位接受监督。

　　各位代表！菏泽所处历史方位的使命担当令人振奋，决胜全面小康实现后来居上的目标前景催人奋进。千万菏泽人民奔康致富的渴求在激励着我们，各级领导对菏泽加快发展的殷切期望在鞭策着我们。让我们更加紧密地团结在以习近平同志为核心的党中央周围，在中共菏泽市委的正确领导下，不忘初心、继续前进，牢记使命、务实图强，全力加快富民强市步伐，为全面建成小康社会、早日实现后来居上目标而努力奋斗！
